# Carbon-Nanotube-Based Monolithic CMOS Platform for Electrochemical Detection of Neurotransmitter Glutamate

**DOI:** 10.3390/s19143080

**Published:** 2019-07-12

**Authors:** Alexandra Dudina, Urs Frey, Andreas Hierlemann

**Affiliations:** 1 Bio Engineering Laboratory, Department of Biosystems Science and Engineering, ETH Zurich, Mattenstrasse 26, CH-4058 Basel, Switzerland; 2 MaxWell Biosystems AG, CH-4058 Basel, Switzerland

**Keywords:** carbon nanotube field-effect transistor, CMOS, ion-sensitive field-effect transistors, ChemFET

## Abstract

We present a monolithic biosensor platform, based on carbon-nanotube field-effect transistors (CNTFETs), for the detection of the neurotransmitter glutamate. We used an array of 9′216 CNTFET devices with 96 integrated readout and amplification channels that was realized in complementary metal-oxide semiconductor technology (CMOS). The detection principle is based on amperometry, where electrochemically active hydrogen peroxide, a product of the enzymatic reaction of the target analyte and an enzyme that was covalently bonded to the CNTFET, modulated the conductance of the CNTFET-based sensors. We assessed the performance of the CNTs as enzymatic sensors by evaluating the minimal resolvable concentration change of glutamate in aqueous solutions. The minimal resolvable concentration change amounted to 10 µM of glutamate, which was one of the best values reported for CMOS-based systems so far.

## 1. Introduction

The integration of 1D nanomaterials, such as carbon nanotubes (CNT), into sensor array platforms yields a sensitive tool for the detection of biological species and it has significant advantages over conventional optical detection methods [1,2]. The first advantage is related to the size compatibility of sensor and analyte: the diameter of a CNT, which is ~1 nm, is comparable to the size of single molecules (e.g., the DNA molecule has a size of 1 nm [1]). The second advantage of CNT-based sensor platforms is that most biological processes involve electrostatic interactions and charge transfer, which can be directly detected by charge-sensitive CNTs. In the case of single-walled carbon nanotubes (SWNTs), every atom is at the surface and it is exposed to the environment and, thus, even small changes in the environment can cause significant changes in the nanotube electrical properties [3,4]. Finally, the material carbon provides a natural match to organic molecules. Thus, among different nanomaterials, carbon nanotubes have a great potential for biosensor applications.

The use of field-effect transistors, based on carbon nanotubes (CNTFETs), has been reported in literature for the electronic detection of single-molecule dynamics, [5,6], highly localized measurements of intracellular electrophysiology, [7], and for label-free detection of disease-related biomarkers and viruses [8,9].

The CNTFET sensing mechanism is similar to that of ion-sensitive FETs. The charge carriers or ions in the liquid phase, located in close proximity to the CNTFETs, lead to an electrostatic potential buildup. This potential depends on the nature and concentration of the charged species and it modulates the electrical resistance of the CNTFETs [3].

It is known that the properties of CNTs depend strongly on their physical characteristics, such as diameter, length, the presence of residual catalyst, and their chirality [10]. For example, CNTs can be either single-walled (SWCNTs) or multi-walled (MWCNTs) with varying intrinsic bandgaps and helicities. In addition, SWCNTs can be either metallic conductors or semiconductors, based on the chirality of the structure [11].

The majority of research efforts towards bio-sensing involves interactions of proteins with CNTs. The attachment of proteins to carbon nanotubes can be performed in several steps after preliminary chemical functionalization of the CNTs. For example, carbon nanotubes can be oxidized to have free carboxyl groups that then undergo coupling with amino groups in proteins [12,13].

Although the various applications of CNTs for the detection of various bio species have been reported previously, the effects of the spread in inherent CNT characteristics, such as semiconducting versus metallic properties or the number of parallel-connected CNTs per sensor, on the device or sensor sensitivity, have yet to be established.

In this paper, we used a complementary metal-oxide semiconductor (CMOS) sensor platform, comprising an array of 9216 CNTFETs, along with readout and amplification channels, for the detection of the neurotransmitter glutamate. A dielectrophoresis-based method (DEP) was used to integrate the CNTs with the electrode array. Given the statistical nature of the DEP deposition process, a large range of CNT-sensor characteristics was observed, as there were single CNTs or bundles of parallel CNTs deposited between the electrode pairs. We characterized the CNTFET ensemble across the whole array in terms of their sensitivity to solutions of different pH-values and showed that the sensitivity was inversely proportional to the initial baseline resistance of the CNTFETs in aqueous medium. Afterwards, we demonstrated a successful functionalization of the CNTFETs with glutamate oxidase and the detection of l-glutamate at concentrations as low as 10 µM for using only one CNTFET and 3 µM for averaging the signals of 62 CNTFETs. Finally, we characterized the CNTFETs across the whole array in terms of their sensitivity to solutions of l-glutamate at different concentrations and we showed the interrelation between the minimal resolvable values of glutamate concentration changes and the minimal detectable pH-change values for the CNTFETs.

## 2. Materials and Methods

### 2.1. Experimental Setup

**Figure 1:**
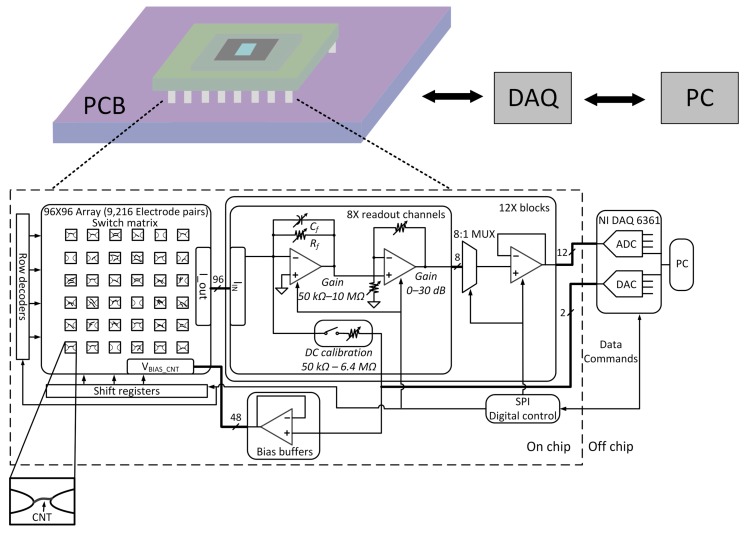
Block-diagram of the overall sensor system and setup (adapted from Reference [14]).

Figure 1 shows a schematic view of the overall system used for this work. The system comprises a monolithic CMOS chip, featuring both, the readout amplifiers and the CNTFET array. The chips were mounted on a custom-designed printed circuit board (PCB), which also provided analog reference voltages and a digital block for bidirectional commination of the chip with a data acquisition card (National Instruments, Munich, Germany).

A novel packaging for the CMOS chip was developed to protect bond wires and readout electronics whilst keeping the electrode array exposed to the liquid analyte solution. After wire-bonding of the CMOS chip to the custom-made PCB carrier, we applied an epoxy encapsulation (EPO-TEK 353, Kummer AG, Cham, Switzerland). A microfluidic chamber, made from PDMS, with a volume of approximately 10 µL was mounted on top of the sensor array. A LabView (National Instruments, Munich, Germany) interface was implemented to maintain communication with the CMOS chip and to transfer the measurement data to a PC. We analyzed the acquired data with custom-made software written in Matlab (Mathworks). Each unit is described in more detail in the following sections.

### 2.2. CMOS Chip

The CMOS readout chip included an array of 9216 platinum electrode pairs and 96 integrated readout and amplification channels. The electrode pairs provided the source and drain connection for the CNTFETs. Two adjacent drain electrodes shared one source electrode. The underlying electrode routing was switch-matrix based, similar to that described in Reference [15].

Each readout channel included two stages of amplification and filtering, followed by a multiplexer (MUX) and a driving buffer. The dedicated digital block, assigned to each readout channel, allowed for independent gain and bandwidth selection. To compensate for the offset and gain error, a resistive feedback loop was implemented in each channel. An offset voltage can add errors to the measurement of the baseline conductance of the sensor, as the bias voltage of the CNT is not precisely defined. The offset compensation was done using six different resistor values in a similar way as reported by Grassi et al in Reference [16]. To define the voltages at the source terminals of each CNTFET, the chip included 48 voltage buffers. Each voltage buffer was an operational amplifier, configured in unity-gain feedback and provided voltages in the range from 200 mV to up to 3.2 V.

**Figure 2:**
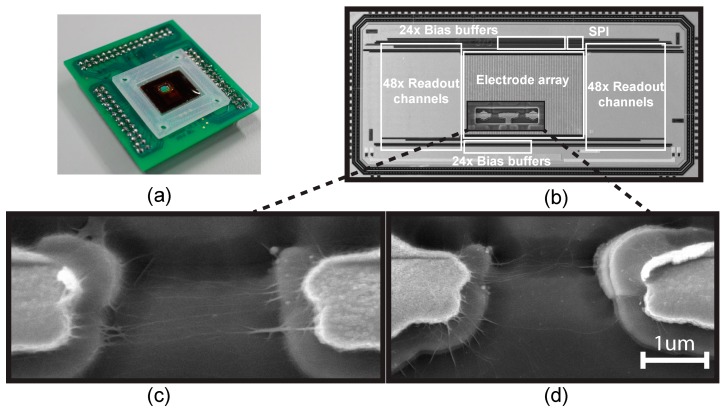
(**a**) Packaged chip; (**b**) Micrograph of a chip die with an area of 6.4 × 3.0 mm^2^; (**c**,**d**) Scanning Electron Microscopy (SEM) pictures showing electrode pairs with integrated carbon nanotubes (CNTs).

The CMOS chip was fabricated in 0.18-μm-CMOS technology, and the overall chip real estate was 6.4 × 3.0 mm^2^. The system was successfully tested and it showed a noise performance of 2.14 pArms at a bandwidth of 1 kHz, and 0.84 nArms at a bandwidth of 1 MHz. Resistances in a range between 50 kΩ and 1 GΩ could be resolved. The chip die is shown in Figure 2.

### 2.3. Fabrication and CNT Assembly

Pairs of platinum electrodes were patterned at wafer level by means of ion-beam metal deposition and etching. A SEM picture of the fabricated Pt electrodes is shown in Figure 2.

To achieve a covalent attachment of proteins onto the sidewalls of CNTs, we used single-walled CNTs (SWCNTs) with carboxylic-acid functionalization (Sigma Aldrich, Buch, Switzerland). These nano-devices had a length of approximately 3 µm and a diameter of approximately 2 nm. The CNTs were assembled on the CMOS chip by means of a dielectrophoresis (DEP)-based deposition technique. The details of the assembly process were described in References [14,17] and were only briefly abstracted in this paper. The initial suspension was prepared by mixing dry CNT powder with distilled MilliQ water, and the mixture was subsequently sonicated for 30 min to obtain a homogeneous suspension. The final concentration of the suspension was approximately 80 μg/L. A wire-bonded, packaged CMOS chip was mounted onto the setup board. The switch-matrix below the electrodes was configured to connect all the electrode pairs in the array to two defined pads of the CMOS chip [14]. An external AC source providing a voltage amplitude of 4 V_pk-pk_ at a frequency of 600 kHz was connected through the pads to the electrode pairs to create an electrical field. The assembly process was carried out for one hour, thereafter the chip was rinsed with deionized water to wash out the rest of the suspension, and it was then gently dried with nitrogen. In Figure 2, one electrode pair with the assembled CNTs is shown.

**Figure 3:**
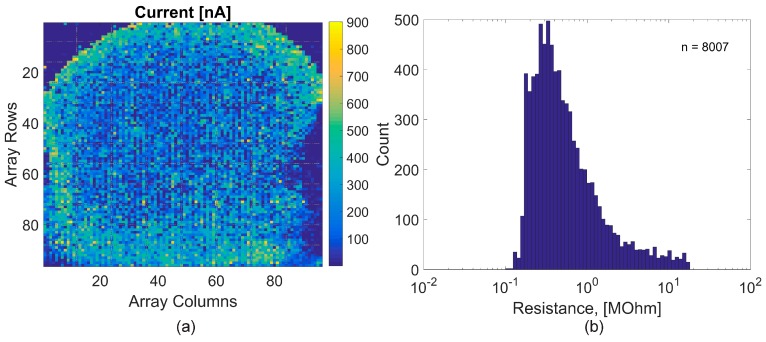
(**a**) Physical map of 8007 CNT-bridged electrode pairs as obtained from electrical conductance tests, (**b**) Histogram of resistance values across the whole array.

Figure 3a represents a physical mapping of the whole array featuring CNTs bridging the electrode pairs. The measurements have been performed in dry air with a bias voltage, V_ds_, of 100 mV. Figure 3b shows the histogram of the corresponding resistances. Owing to imperfections of the packaging process, the boundary region at the top was covered with epoxy and, therefore, it was not available for the assembly process.

### 2.4. Functionalization Protocol

**Figure 4:**
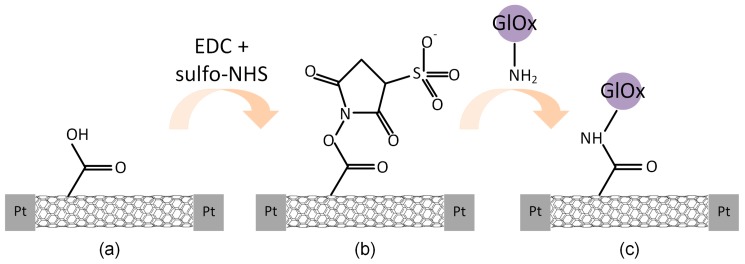
Illustration of the functionalization steps with the corresponding enzymes: (**a**) CNTs featuring carboxylic groups at the surface; (**b**) intermediate stage after the EDC and sulfo-NHS coupling; (**c**) final state after coupling with glutamate oxidase.

All chemicals were purchased from Sigma Aldrich, Buchs, Switzerland. They included 1-ethyl-3-(3-dimethylaminopropyl) carbodiimide (EDC), *N*-hydroxysulfo-succinimide (sulfo-NHS), l-glutamate oxidase from Streptomyces, Tris HCL, Bovine Serum Albumine (BSA), and l-glutamic acid. The CNT functionalization procedure was adapted from Reference [13] and it is briefly illustrated in Figure 4. A CNT array was immersed in a freshly prepared 3-mL aqueous solution of EDC (10 mg/mL), see Figure 4a. Under stirring, 90 mg of sulfo-NHS was added to the solution. The reaction was allowed to proceed at room temperature for 2 h, Figure 4b. Glutamate oxidase (GlOx) was dissolved at a concentration of 2 mg/mL in a filter-sterilized phosphate buffer solution (PBS) with a pH of 8. The volume of the prepared sample was 3 mL. An array chip was washed quickly with MilliQ water and immediately immersed into the prepared solution. The enzyme immobilization reaction occurred at room temperature over 1 h, Figure 4c. After that, the electrode surface was washed with a phosphate buffer solution (pH 7.4) and further incubated for 30 mins in 0.1 M Tris HCl in PBS. The CNTFET surface was washed with PBS and then, the electrodes were blocked with 0.5% BSA in PBS of pH 7.4 for 1 h. Finally, the CNTFET surface was washed one more time in PBS and prepared for the biological experiments.

## 3. Results and Discussion

### 3.1. Sensor Characterization

First, the current−voltage (IV) curves of the CNTFET sensors connecting the electrode pairs were recorded. The sensor characterization was performed before the attachment of enzyme, i.e., the CNTs only had carboxylic groups on the surface. The measurements were performed in a phosphate-buffered saline (PBS, pH = 7.4), (Gibco, Life Technologies, Zug, Switzerland), diluted ten-fold in MilliQ water (10 mM PBS). Using diluted PBS, the ionic strength of the liquid solution could be reduced, which increased the Debye screening length (i.e., the distance at which the surface potential of the CNTFET sensor was completely screened, which resulted in zero response to analytes in solution) [18]. The source-drain voltage was set to 100 mV, whilst the electrolytic gate potential was swept from −400 mV to 250 mV with respect to the source potential.

**Figure 5:**
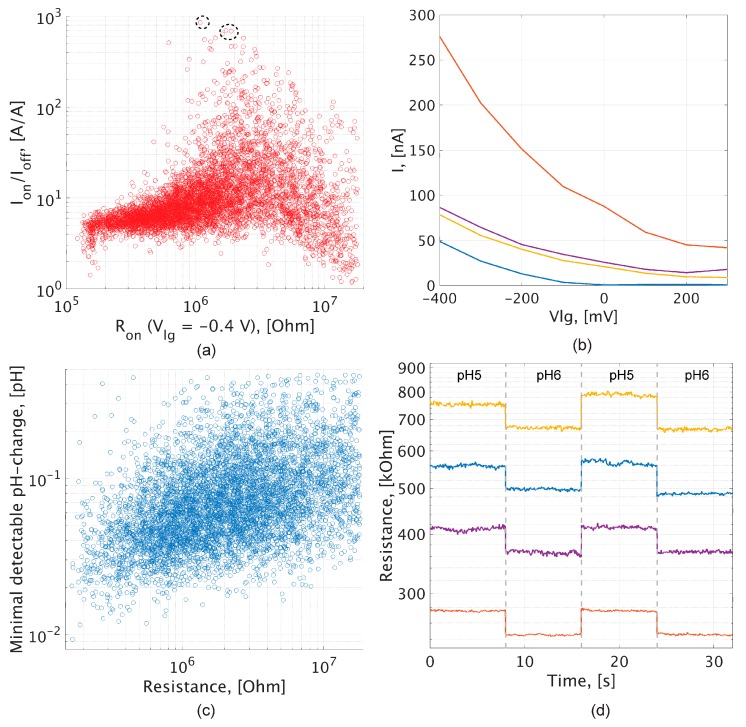
(**a**) Scatter plot of the I_ON_/I_OFF_ ratio versus the R_ON_ of all carbon nanotube field-effect transistors (CNTFET) devices of an array. (**b**) Current–voltage characterization (I-V curves) of CNTFETs with the largest I_ON_/I_OFF_. (**c**) Scatter plot of minimal detectable pH-changes versus R_PH5_; (**d**) Resistance changes versus time of 4 different CNTFET devices upon sweeping the pH values from 5 to 6 two times.

The values of the drain current at a gate potential of −400 mV and 250 mV were defined as I_ON_ and I_OFF_. The value of the CNTFET resistance at a gate potential of −400 mV was defined as R_ON_. Figure 5a shows the plot of the ratio of I_ON_/I_OFF_ values of each CNTFET device versus R_ON_ over the full array. These values can be used to deduce the nature of the CNT devices (semiconducting or metallic). The devices featuring higher resistances were considered to be semiconducting CNTs and show a higher I_ON_/I_OFF_ ratio, whereas the devices with lower resistance were considered to be metallic or bundles of CNTs of mixed type.

Figure 5b shows the I-V curves of CNTFETs featuring the highest I_ON_/I_OFF_ ratio, marked by the dashed circle. The CNTFETs appeared to behave like p-type field-effect transistors, exhibiting an overall electrical conductance decrease upon sweeping the potential of the reference electrode from negative to positive values with respect to the source potential.

We performed a CNTFET sensitivity analysis, namely the estimation of the minimal detectable pH-change value across all CNTFETs over the whole array. Sodium phosphate buffer solutions with two different pH-values were prepared. The solutions of pH-values 5 and 6 were applied sequentially to the microfluidic chamber hosting the CNTFET array. V_ds_ was set to 100 mV, the electrolytic gate potential was set to −300 mV. The measured data were recorded by simultaneously sampling 96-channels at 4 kHz and then sequentially scanning the whole CNTFET array. The response of every CNTFET sensor was recorded over 8 s.

The acquired raw data were low-pass filtered with a corner frequency of 100 Hz. The estimation of the minimal resolvable pH-change value was based on a calculation of the Fisher information (statistics). The Fisher information describes the variance of the mean value of the measured experimental dataset. Finally, the resolution was defined, based on the Cramer Rao bound, which is the inverse of the square root of the Fisher information. Figure 5c shows the scatter plot of the minimal detectable pH-change value of individual CNTFETs at pH = 5, R_pH5_, is shown versus their baseline resistance. The best-achieved value was as low as 0.01 pH units for individual CNTFETs. From the figure, the sensitivity of the CNTFETs was inversely proportional to the initial resistance of the CNTFETs, so that the sensitivity of devices with lower resistance was higher than the devices featuring higher resistance. This finding could be explained by the fact that the pH sensitivity was based on the protonation or deprotonation of the functional groups at the surface of the CNTFETs. At lower pH values, (pH 5), the carboxylic groups get protonated, which provides positive charges on the gate, whereas, at higher pH values, the carboxylic groups get deprotonated, which provides negative charges on the gate. The slope of the I-V curve, however, depends on the formation of a double layer and the accumulation of positive (Na^+^) or negative (Cl^−^) charges at the surface of the CNTFET devices, which depends on the potential that has been set at the reference electrode. It is likely that CNTFETs featuring lower resistance values feature bundles of CNTs that are connected in parallel between the electrode pair. A bundle of CNTs has more functional groups at the CNTFET surface, which contribute to the change of the sensor resistance upon changing the solution pH. Figure 5d shows the pH traces of four individual CNTFETs versus time. The minimal resolvable pH change for these sensors amounted to approximately 0.01 pH-units. The CNTFETs showed a good stability and a very small drift.

### 3.2. Detection of Neurotransmitters

Glutamate is an excitatory neurotransmitter, which is released at synaptic terminals [19]. It plays a major role in various neural functions and relates to various neurological disorders, as well as to memory and learning processes [20]. For example, an excessive release of glutamate plays a key role in neuronal death associated with a wide range of neural disorders [20]. Therefore, real-time monitoring of extracellular glutamate levels would be very helpful in understanding the excitotoxic process of neurotransmitters in the case of brain injury.

**Figure 6:**
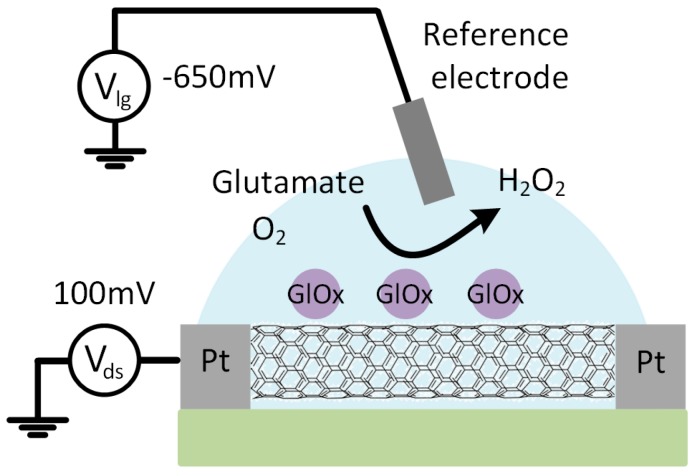
Illustration of the working principle of enzyme-functionalized CNT-based biosensors.

Biosensors for glutamate are usually based on glutamate oxidase (GlOx). The working principle is illustrated in Figure 6. The GlOx catalyzes the oxidation of glutamate to 2-oxoglutarate in the presence of oxygen, which produces electrochemically active H_2_O_2_.

Glutamate + O_2_ → α-ketoglutarate + NH_3_ + H_2_O_2_ (1)

Quantification of glutamate by means of amperometric biosensors is achieved via electrochemical oxidation of the liberated H_2_O_2_ [19,21]. The oxidation of H_2_O_2_ requires application of a potential of -0.65 V to the reference electrode. The concentrations of glutamate in extracellular recordings in neuronal preparations are in the range between a few µM to up to few tens of µM [19].

All solutions were freshly prepared and kept at room temperature. All experiments were carried out at room temperature. The enzymes were attached to the carboxylic functional groups of the CNTs using standard protocol of EDC and sulfo-NHS coupling, namely, forming amide linkages between the enzyme amine residues and the carboxylic-acid groups of the CNTFETs [12]. The functionalization protocol has been described in the Materials and Methods section.

**Figure 7:**
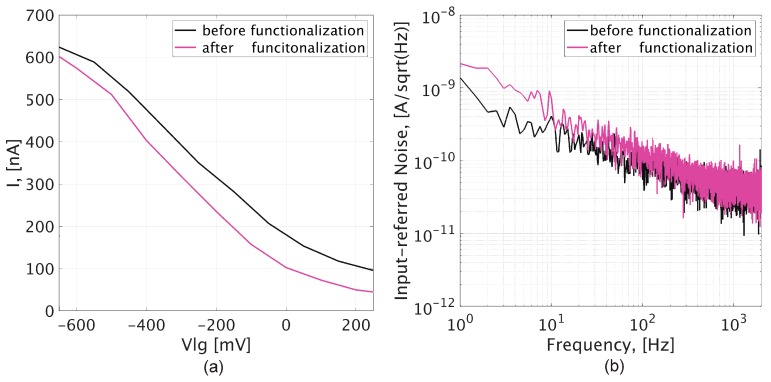
(**a**) Current–voltage characterization (I-Vlg curve) of a CNTFET before (green) and after (red) enzyme immobilization; (**b**) Input-referred power spectral density (PSD) of the corresponding CNTFET device, recorded in phosphate-buffered saline (10 mM PBS).

**Figure 8:**
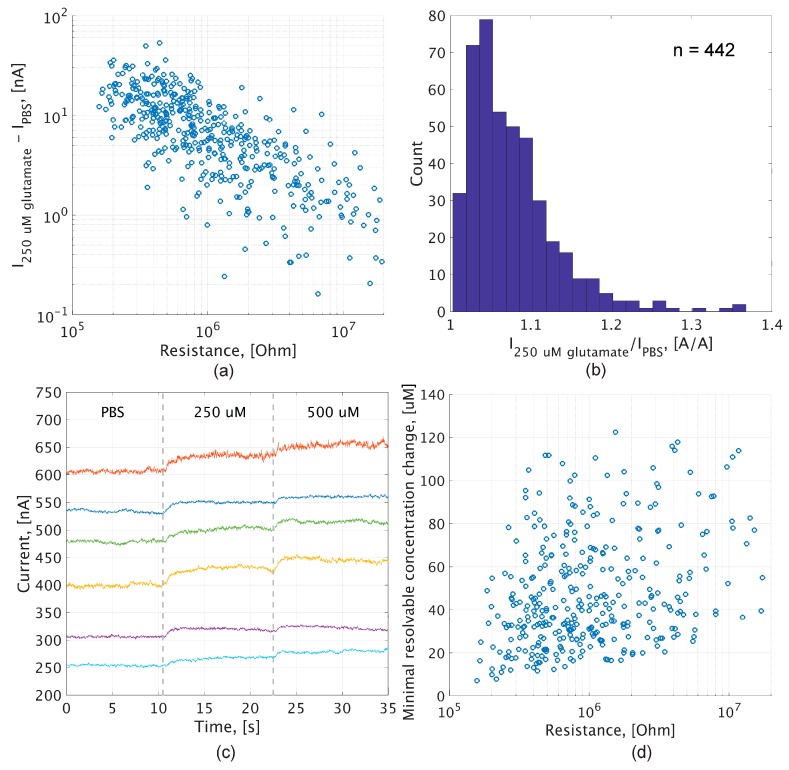
(**a**) Scatter plot of the absolute changes of the CNTFET currents, ∆I = I_250µM___glutamate_ − I_PBS_ versus the respective initial resistance values, R_PBS_; (**b**) Histogram of the respective relative current changes of 442 CNTFETs of the array; (**c**) Amperometric curves versus time of 6 different individual CNTFET devices upon applying glutamate solution of two different concentrations; (**d**) Scatter plot of the minimal resolvable values of glutamate concentration changes versus the respective initial resistance values, R_PBS_.

Figure 7a shows the I-V curves of one randomly selected CNTFET, measured before and after functionalization. The measurements were done in PBS (10 mM). During the measurements, the bias voltage of the CNTFET was set to 100 mV, whereas the potential at the reference electrode was swept from -400 mV to 200 mV. We observed a decrease in conductance of the CNTFETs upon GlOx attachment, which could be explained by the effect of a positive charge introduced to the CNTFET surface [22,23]. An adsorption of a positively charged species induces additional negative charges in the CNTFET, thus n-doping of the CNT and a shifting of the I-Vlg curve towards more negative gate voltage [3]. Figure 7b shows the power spectral density (PSD) of the CNTFET before and after functionalization. During the noise measurements, the potential of the reference electrode was set to 0 V. The enzyme, attached to the surface of CNTFET, introduced additional noise, mostly in the low-frequency range [24].

l-glutamic acid was dissolved in 10 mM PBS at different concentrations. The solutions were freshly prepared and kept at room temperature during the experiments. The amperometric response to glutamate for two concentrations of 250 µM and 500 µM was recorded under steady-state conditions by applying a potential of −650 mV to the Ag/AgCl reference electrode. The CNTFETs were biased at 100 mV. We defined the resistance value, R_PBS_, at 10 mM PBS as the reference value. In Figure 8a, the absolute ratio of current change, ∆I = I_250µMo_ − I_PBS_, is plotted versus the respective resistance values, R_PBS_. We observed that the current changes of the CNTFET devices upon an increase in the concentration of the analyte solution depended on the initial resistance of the CNTFET devices, and that the ∆I -ratio values ranged between 48 nA and approximately 160 pA. The CNTFET devices with lower initial resistance exhibited higher absolute current changes. A hypothesis to explain this finding was that the sensitivity depended on the number of functional groups to which the enzyme was attached. CNTFETs with lower resistance values were comprised of bundles of CNTs, which feature more functional groups, so that more enzyme molecules could be attached to those groups and were available for the analyte-detection reaction. Figure 8b shows the distribution of the relative CNTFET current change ratios, I_250µMo_/I_PBS_. The average value amounted to 1.07. The amperometric responses of six different CNTFETs upon sweeping the concentration of glutamate is presented in Figure 8c. The sensors showed a linear behavior for the measured concentrations of 250 and 500 µM. The response time of the individual CNTFETs to a change in the solution concentration was approximately 4 s. A scatter plot of minimally resolvable glutamate concentration changes versus initial sensor resistance, R_PBS_, is shown in Figure 8d. The estimation of the values was performed in a similar way as in the case of the minimal resolvable changes in pH-values by calculation of the Fisher information and the Cramer Rao bound as the inverse of the square root of the Fisher information. From the plot, the CNTFETs with lower initial resistance showed higher sensitivity. The best achieved value was 10 µM of glutamate concentration change.

**Figure 9:**
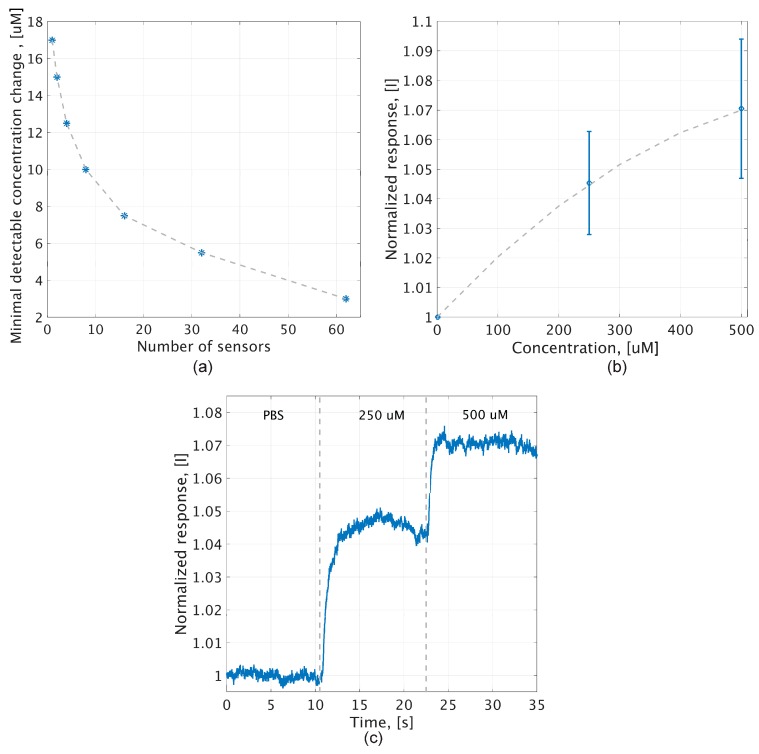
(**a**) Scaling of the minimal resolvable l-glutamate concentration with the number of used CNTFET devices; (**b**) Calibration curve of 62 devices. The relative non-linearity amounted to 3%; (**c**) Normalized, averaged current response, [I], of 62 CNTFET devices upon applying l-glutamate solutions of different concentrations.

We also studied how the minimal resolvable concentration change values scale with the number of used CNTFETs. In Figure 9a, we plot the scaling of the minimal resolvable l-glutamate concentration change value with the number of used CNTFET sensors from one randomly selected block. The best achieved value was 3 µM of glutamate upon averaging 62 CNTFETs out of 96 in one randomly recorded block. The other 34 CNTFETs in this block did not show any response, or there was no CNT connecting the electrode pair. The averaging of the signals of 62 CNTFETs gave approximately a 5.6 times relative improvement of the minimal resolvable concentration change value. We observed a similar trend in the measurement data recorded from another randomly selected block.

The calibration curve of the averaged normalized current responses of 62 CNTFETs over the concentration range between 250 µM and 500 µM is shown in Figure 9b. The curve shows an approximately quadratic dependence on the analyte concentration. Figure 9c shows the averaged normalized amperometric curve (normalization to the initial current, when only PBS solution has been applied to the CNTFET sensor array) of 62 CNTFET devices for the two applied analyte concentrations.

Table 1 presents a comparison of the performance of the designed system with systems reported in other references.

**Figure 10:**
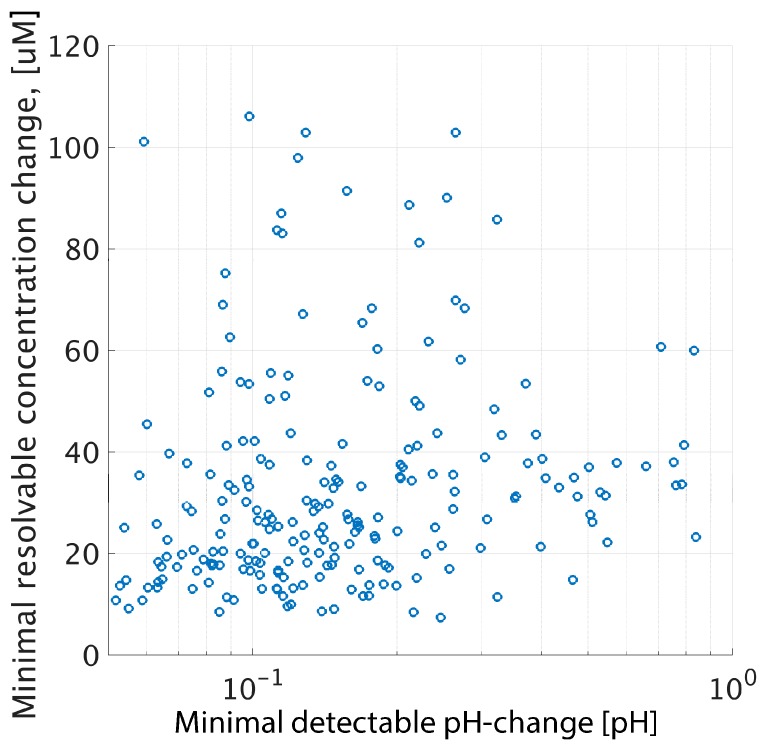
Scatter plot of minimal resolvable values of glutamate concentration changes versus the minimal detectable pH-change values.4. Conclusions

A scatter plot of minimally resolvable glutamate concentration changes versus the minimal detectable pH-changes value is shown in Figure 10. From the figure, the CNTFETs featuring better sensitivity to pH-change values show a higher sensitivity to glutamate concentration changes. This finding could also be explained by the fact that the pH sensitivity, as well as the detection of the enzyme, is based on the chemical reaction of the functional carboxylic groups on the CNTFETs surface either with ions in the solution or with the target enzyme. The method to characterize the sensitivity of CNTFETs with different pH buffer solution may potentially be applied to estimate the sensitivity and the performance of the fabricated sensor array featuring the target enzyme or to fabricate the sensor arrays with graded sensitivity.

We presented a monolithic CMOS system for electrochemical detection of the neurotransmitter glutamate. The system features a large array of CNTFETs, as well as 96 tunable readout and amplification channels. The system sensing functionality was verified through the measurement of CNTFET responses to buffer solutions of different pH values. The performance of the CNTFETs as pH sensors was assessed by determining the minimal resolvable pH-change. The best achieved value was as low as 0.01 pH units. It was demonstrated that the sensitivity of the CNTFETs was inversely proportional to their initial baseline resistance. A successful functionalization of the CNTFETs with the enzyme glutamate oxidase and the detection of l-glutamate was demonstrated. The performance of the CNTFETs as enzymatic biosensors was assessed by evaluating the minimal resolvable concentration change of the corresponding analyte. The system was able to detect concentrations within the physiological range. The best achieved values amounted to ~10 µM of l-glutamate for individual CNTFETs, whereas for several averaged CNTFETs, this value was as low as 3 µM of l-glutamate.

The achieved results demonstrate the potential of CNT-based monolithic sensor platforms. The variation and control of the parameters of the assembly process will provide the potential to fabricate sensor arrays with graded sensitivity, which can be useful for various biomedical applications.

## Figures and Tables

**Table 1:** Comparison of the performance of the designed system with the other references.

Parameter	[19]	[25]	This work
System	Passive electrodes	CMOS chip	CMOS chip
Number of CNT sensors	1	64	9′216
Number of channels	1	1	96
Limit of detection	5 µM	10 µM	10 µM (3 µM for 62 CNTFETs)
